# Time course of perceived knee stiffness following prolonged sitting in healthy adults

**DOI:** 10.1016/j.jsampl.2025.100124

**Published:** 2025-12-01

**Authors:** Alexandre Kovats, Jeanette M. Thom

**Affiliations:** a School of Health Sciences and Sydney Musculoskeletal Health, The University of Sydney, Australia; b School of Health Sciences, UNSW Sydney, Australia

**Keywords:** Knee joint, Knee pain, Sedentary behaviour, Range of motion

## Abstract

**Background:**

Knee stiffness is a common complaint experienced during prolonged periods of sedentary behaviour, including following prolonged sitting, in both healthy and people with musculoskeletal conditions. Reduction of self-reported knee stiffness is important to patient-centred outcomes. Our aim was to obtain pilot data to determine the timeframe for knee stiffness onset in healthy populations.

**Methods:**

Forty-two participants (20–76 years) with no diagnosed chronic musculoskeletal condition were recruited. After pilot testing, perceived knee joint stiffness and pain (Visual Analogue Scale) were measured while sitting (at 15, 30, 60, and 90 ​min) with the knee joint held at 90°. Correlations between knee stiffness after 90 ​min of sitting and baseline demographic data, and knee range of motion were analysed.

**Results:**

Greater levels of self-reported stiffness were observed (mean ​± ​SD) after sitting still for 30 (1.8 ​± ​1.6; p ​< ​0.001), 60 (3.4 ​± ​2.4; p ​< ​0.001) and 90 ​min (4.1 ​± ​2.6; p ​< ​0.001) compared to baseline (n ​= ​31). Increase of self-reported pain occurred by 60 and 90 ​min of sitting (p ​< ​0.001). Minimal clinical important difference (MCID) (±2 ​cm) in stiffness occurred by 60 ​min of sitting (+2.6 ​cm) with pain not reaching MCID by 90 ​min. Self-reported stiffness at 90 ​min of sitting was negatively correlated with participant age (r ​= ​−0.433, p ​= ​0.004).

**Conclusions:**

Healthy adults begin to experience knee stiffness when sitting still after 30 ​min, though this was only clinically relevant by 60 ​min. Moving the joint, even whilst still sitting, helped to alleviate joint stiffness and pain, which may assist in providing advice to all adults on sedentary behaviours.

**Key points:**

• Healthy adults experience knee joint stiffness after sitting still for only 30 ​min, but this stiffness only reaches more meaningful levels after 60 ​min. Knee joint pain is also observed after sitting still, but this remains at a low level.
• It only takes less than a minute of moving the leg, even whilst still sitting, for perceived knee joint stiffness (and pain) to decrease, though after 90 ​min of sitting this is still higher than baseline levels.
• Older adults are less likely to perceive sitting still for long periods of time to increase levels of their knee joint stiffness and pain.

## 1 Introduction

Since the seminal study of the health of London bus drivers and conductors [1], the importance of physical activity (PA) in comparison to long bouts of sedentary behaviours, especially prolonged sitting, has been highlighted. Nowadays, a fundamental component of adult health guidelines [2] are the promotion and inclusion of PA and decreasing sedentary behaviours. However, we currently have an increasingly sedentary workforce and engage in more recreational sedentary behaviours. With half of Australian workers reporting that they sit often or all the time at work [3]. Thus, not only is there is an increasing need for promotion of active lifestyles globally and across all age groups, but there is also a requirement to investigate the deleterious effects of increased sedentary activity.

The negative health effects of prolonged sitting, including increased musculoskeletal disorders and cardiometabolic conditions [[2], [3], [4], [5], [6], [7], [8], [9], [10], [11]], have been observed in a range of populations, e.g. office workers [8,12], occupational drivers [[13], [14], [15], [16]], and students [17]. Whereas, the benefits of breaking up bouts of prolonged sitting has been observed [2,[9], [10], [11]], and includes decreased muscle stiffness [9] and improved cardiometabolic outcomes [7], fatigue and maintaining work productivity [11].

Importantly, musculoskeletal stiffness and discomfort is not only experienced by individuals with pathology, but it also occurs regularly in the general population with prolonged bouts of sitting [8,9,18]. However, there is currently limited data on the time course knee joint stiffness with prolonged sitting. Knee stiffness is a common patient reported complaint experienced by people with apparently healthy knees as well as musculoskeletal pathologies, specifically during prolonged periods of sedentary behaviour (i.e. prolonged sitting) [4,5]. Joint stiffness can be an early indicator of osteoarthritis (OA) [19], and has been reported to occur in 31–50 ​% of people with knee OA [20,21].

Given the progressive nature of musculoskeletal pathologies, such as OA, and the increasing rates of adult sedentary behaviour, it is important to investigate options to the reduce self-reported symptoms of joint stiffness. A fundamental component of musculoskeletal pathology evidence-based care is the promotion and inclusion of PA and decreasing sedentary behaviours [4,5,22,23]. When correctly performed, acute bouts of movement, decreasing weight and a range of exercise types have been shown to positively affect cartilage and synovium health, decrease pain and improve overall joint mobility and function [[23], [24], [25]]. Although undertaking PA to improve stiffness and other musculoskeletal symptoms has been reported to be a facilitator, having joint stiffness, along with pain and fatigue, is also a barrier to engage in PA [26].

Although the reduction of self-reported musculoskeletal symptoms is of paramount importance to people after prolonged sitting, there are several aspects that require clarification. One is that joint stiffness is difficult to quantify [27]. For example, in OA, self-reported joint stiffness may be distinct from mechanical stiffness [28]. Moreover, studies investigating prolonged sitting have focused on muscle stiffness rather than stiffness observed in joints. In a control-matched investigation of joint stiffness following 20-min of sitting, people with patellofemoral pain (PFP) observed increased stiffness, whereas, healthy knee participants reported no significant change [29]. Thus, there is still no definitive time course for knee stiffness to occur in healthy following prolonged sitting.

The primary aim of the current study was to determine the time of onset for perceived knee stiffness in healthy adults during prolonged sitting. Secondary aims of this study included the determination of the time course of pain, the recovery of both pain and stiffness following prolonged sitting and if perceived stiffness following sitting is correlated with demographic and baseline functional variables.

## 2 Methods

### 2.1 Study design

**Fig. 1:**
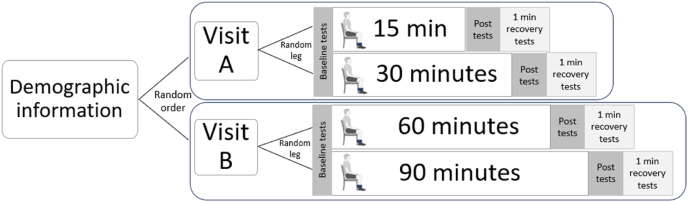
Graphical description of the prolonged sitting protocol. Participants were randomised to visit order and to which limb was strapped to the chair for the longer period in each session (either Visit A: 15 or 30 ​min or Visit B: 60 or 90 ​min). Outcome measures were recorded, at baseline, immediately post sitting and 1 ​min post the leg strap being removed (whilst still seated). Note that the participants were able to stand up from the chair after the limb tested for the longer time in each visit (e.g. after 30+ minutes for visit A).

Study design utilised a single arm randomised cross-over design (by limb and visit) with repeated measures observing change in knee joint stiffness following prolonged sitting. For participant comfort and efficiency of time (i.e. only requiring two testing sessions and not four), both limbs were used. This was made possible by randomising the four time points, 15, 30, 60 and 90 ​min between the left and right limbs across two separate occasions, refer to Fig. 1 (e.g. Visit A: Right limb measured after 15 ​min and left limb after 30 ​min, and Visit B: Right limb measured after 60 ​min and left limb after 90 ​min). Any differences in pain or stiffness that participants may have had between limbs were thus counteracted by the randomisation of limbs and visit order between participants.

The research was conducted at UNSW, Sydney. Ethical approval was obtained by the UNSW Human Research Ethics committee (HC16557), and the study was undertaken in accordance with the Declaration of Helsinki.

Written consent outlining the study was provided via email or in person from all participants, with completed consent forms collected prior to commencement of the research study. To maintain confidentiality all physical and electronic data was deidentified and stored in a secure location at UNSW once collected.

### 2.2 Participants and recruitment

The inclusion criteria were healthy participants over 18 years old, with a body mass index (BMI) ​< ​28 ​kg/m^2^ and able to attend the campus on two visits. Participants were excluded if they had any diagnosed chronic conditions (i.e. OA, rheumatoid arthritis, or PFP), recent acute conditions (i.e. joint injury or fracture), deep vein thrombosis or varicose veins, pregnancy and/or had performed high intensity activities in the previous 48 ​h.

Participants were recruited locally through emails, locally placed advertisements, or via word of mouth and via associated approved social media (e.g. UNSW-related Facebook groups).

### 2.3 Outcome measurements

*The primary outcome* was perceived knee joint stiffness using a visual analogue scale (VAS) at multiple time points (15, 30, 60, and 90 ​min). *Secondary outcomes* were perceived knee joint pain via VAS at the same time points; knee stiffness and pain scores obtained in recovery at 1-min after each of these time points, whilst the participants remained seated but were able to move their lower limb freely; and correlations between stiffness at 90 ​min of sitting with baseline demographic measurements and knee function.

Participants were asked to complete a paper-based VAS to self-report perception of both knee joint stiffness and pain on a 10-cm scale, a method which provides reliability to ±0.2 ​cm [30]. For each time point, participants were handed a blank VAS, being blinded to previous results. Pain was recorded first, whilst sitting with the knee in approx. 90° of flexion with the foot on the floor. Knee stiffness was recorded with the participants remaining seated while they were instructed to move their lower limb freely, as stiffness is described as ‘a sensation of restriction or slowness in the ease with which you move your knee joint’ [31]. These measurements occurred prior and immediately after the period of sitting. Knee stiffness and pain scores were also obtained whilst the participants were still seated at 1-min after each of the bouts of prolonged sitting time points.

To determine at what time the self-reported pain and stiffness following prolonged sitting were meaningful, the participants values were compared to minimum clinically important difference (MCID) values. Change by a MCID, when recording self-reported scores, indicate the patient perceived effectiveness of an intervention and thus are an effective tool linking research to practice [32]. A MCID for pain VAS rating requires a change in 1.99 ​cm or greater to be considered meaningful for patients with knee OA; while no such MCID appears to exist for stiffness via VAS, other measures have a similar 20/100 estimate [33,34].

At baseline, demographic (age, height and weight), basic medical history and PA data were collected. Self-reported medical history was used to reconfirm if the participants had any exclusion criteria and to determine their relative risk of participant during sitting (i.e. cardiovascular DVT or recent injury) and excluded from the study if the met any of these criteria. Knee Osteoarthritis Outcome Score (KOOS) assessed patient reported symptoms, stiffness, pain, functions of daily living, and sport/recreational function [31]. The primary KOOS question of interest was the Stiffness subscale that uses questions “How severe is your knee joint stiffness after first wakening in the morning?” and “How severe is your knee stiffness after sitting, lying or resting later in the day?” (Scores range 0 (none) to 4 (extreme), subscale total ​= ​8). The use of individual or sub-groups of items from the KOOS has previously been reported [[35], [36], [37]]. The Physical Activity Scale for the Elderly (PASE) questionnaire was administered to determine PA over the past week, with higher scores indicating greater PA (Scores range 0–793; average score 131 ​± ​70 in sedentary adults of mean age 66.5 ​± ​5.3 years) [38]. This PA questionnaire was chosen due to its common use across a range of populations [39].

Prior to the prolonged sitting protocol several baseline measurements were obtained on both limbs and averaged (no difference between limbs in any outcome was observed, p ​> ​0.05). Knee passive range of motion (ROM) was measured with a JAMAR™ goniometer (JLW Instruments, Chicago, USA) around the sagittal plane of the knee joints (greater trochanter to lateral femoral condyle and lateral femoral condyle to lateral malleolus) to determine maximum flexion whilst the participant was lying prone on a plinth.

### 2.4 Pilot protocol

**Fig. 2:**
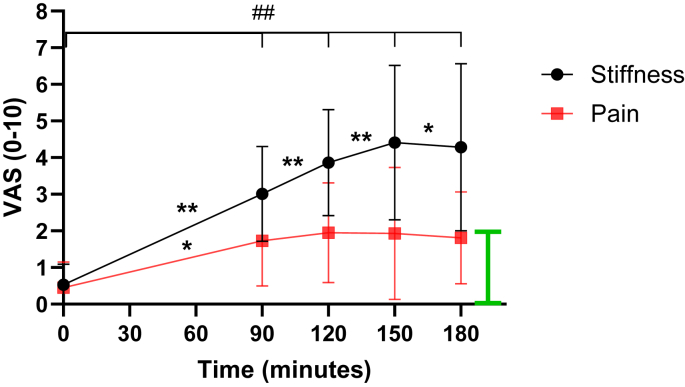
Pilot study with 11 participants showing the time course of self-reported knee joint A) stiffness and B) pain via visual analogue scale (VAS) for up to 180 ​min of sitting with knees held at approx. 90°. Highlighting that significant changes had occurred within 90 ​min of prolonged sitting. Data are means and 95 ​% CI. ## Denotes difference from baseline at all timepoints, pain: p ​< ​0.05, stiffness: p ​< ​0.001. Difference from previous time point for both stiffness and pain: ∗p ​< ​0.05 ∗∗p ​< ​0.01. **Green bar** denotes Minimal Clinical Important Difference from VAS ​= ​0.

Prior to the study, pilot testing of the minimum length of time that participants were required to remain seated was undertaken with n ​= ​11 volunteers. These participants completed the study as described below but remained seated for up to 180 ​min (90, 120, 150 and 180) minutes). Data from this pilot study (Fig. 2) determined that a meaningful change in knee stiffness had already occurred and that pain had plateaued by 90 ​min. Thus, the main study protocol time period was adjusted to a maximum of 90 ​min. These 11 participants are included in the associations of knee stiffness (at 90-mins) with baseline variables.

### 2.5 Main study protocol

**Fig. 3:**
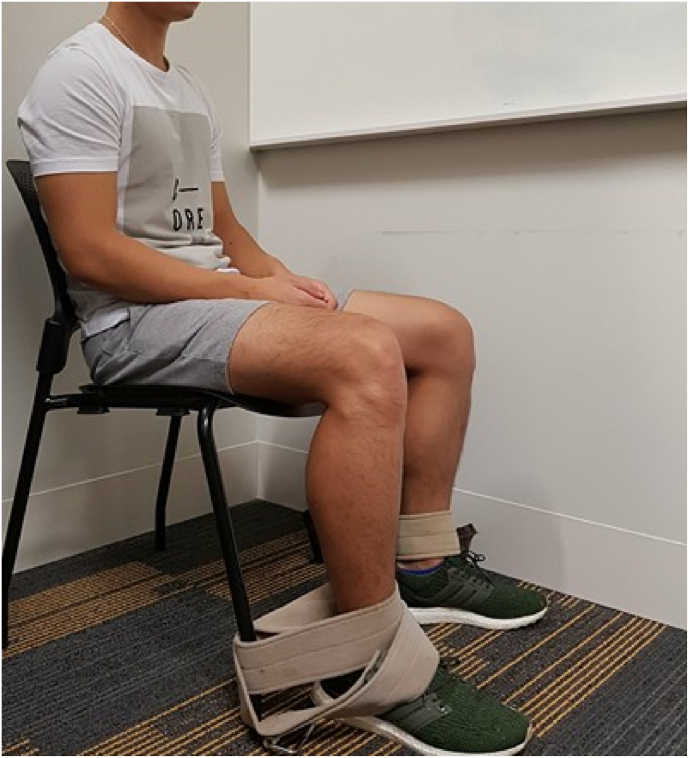
Standardised participant sitting position at approx. 90° knee flexion with Velcro straps loosely attached around the ankles to minimise lower limb movement.

At each of the two visits and after baseline measures were completed, the participants were verbally instructed to be seated in a standardised chair with their foot position adjusted so that the knee joint was at approx. 90° of flexion. Participants remained in this position for the remainder of their time condition (Visit A: 15 ​min or 30 ​min or Visit B: 60 ​min or 90 ​min) with their ankles secured to the chair legs by Velcro straps to maintain the immobilised position of the knee (Fig. 3). The length of time each limb was tested was determined by the study randomisation. Both knees were tested at two individual time points each over the two visits, resulting in a total of four repeated time point measurements for both stiffness and pain (Fig. 1). The time points of data collection occurred at 15, 30, 60 and 90 ​min.

Visit and leg order allocation for each participant was randomised via Microsoft Excel randomisation function by the chief investigator at the start of the project, prior to participants being recruited. The chief investigator held the participant concealment mechanism and randomisation code on a secure computer and provided the relevant participant order to the investigator on the test day of each participant. Blinding of participants was not applicable.

Immediately post each of the protocol time points, one of the participants limbs was unstrapped and the participant was instructed to provide a self-reported VAS rating of pain before being instructed to freely move the knee joint through flexion and extension to provide a VAS rating on stiffness. Following both legs finishing their allocated time at each visit, participants were instructed through a short exercise session of light intensity, including ROM body weight for the lower limbs and walking for up to 15 ​min. Additionally, participants were provided information regarding signs of DVT and a list of exercises that they could do whilst sitting in future, similar to those provided on long-haul flights.

Following the initial visit, participants were asked to return on another day at a similar time of day for the remaining time conditions where the sitting study protocol and outcome measures were repeated.

### 2.6 Study size

There was no existing data that encompassed joint stiffness from prolonged sitting in healthy adults for over 20 ​min. Power calculation was performed via difference between means from younger adults with PFP pre and post 20-min sitting [29], *α* ​= ​0.05, 1-*β* ​= ​0.95 using G.Power 3.1.9.7. This calculated a minimum sample population size at 11 participants for stiffness (pre 2.68 ​± ​2.24 to post 4.93 ​± ​1.76; mean ​± ​SD; effect size ​= ​1.10), or 14 participants for pain (pre 1.46 ​± ​1.87 to post 3.21 ​± ​1.87; effect size ​= ​0.94). To allow for dropouts, the fact that our cohort encompasses a greater age range and will be sitting for longer (up to 1.5 ​h) and that they do not have PFPS, the current study aimed to recruit at least 20 participants across a range of ages.

### 2.7 Statistical analysis

Descriptive statistics, sample means, and standard deviation for primary and secondary outcomes were performed (Windows SPSS v28.0). For the pilot (n ​= ​11) and main study (n ​= ​31) the analysis of the primary outcome (*Stiffness*) and secondary outcome (*Pain*) employed a simple Linear Mixed Model analysis across the five time points, with parametric Bootstrapping and Bonferroni-adjusted post-hoc comparisons after checking for residual diagnostic plots for normality and constant variance. Where assumptions were not met (Pain) data were transformed on the log10 scale. Pearson's correlations were employed to determine the relationships of secondary outcomes (age, PA, KOOS (symptoms, pain and stiffness scores) and ROM) with stiffness at 90 ​min of sitting (n ​= ​42), as this time point corresponded to the sitting time where all participants could be included across the pilot and main study. Data presented as means ​± ​SD, unless specified and significance level for the study was set as p ​< ​0.05.

## 3 Results

Forty-two (n ​= ​42) participants between the ages of 20 and 76 with no diagnosed chronic and low modifiable risk factors for musculoskeletal disease volunteered for this study (see Table 1). Eleven participants completed the pilot study and 31 participants the main study. There were no significant differences between the two groups in baseline characteristics and thus all 42 participants were used to correlate both stiffness and pain after 90 ​min of sitting with the baseline variables. There were also no differences between right and left limbs in baseline measures (data not shown). Normality of the data was met, and the constant variance was approximately met.

**Table 1:** Participant characteristics and baseline outcome measures.

Outcome measure (mean ​± ​SD unless stated)	Pilot study	Main study	All participants
	N ​= ​11	N ​= ​31	N ​= ​42
Sex (M:F)	5:6	16:15	21:21
Age (years)	40.0 ​± ​20.0	30.5 ​± ​13.6	33.0 ​± ​15.8
	20–76 years	20–61 years	20–76 years
Height (m)	1.67 ​± ​0.10	1.70 ​± ​0.10	1.70 ​± ​0.10
Weight (kg)	68.3 ​± ​13.5	67.2 ​± ​12.9	67.5 ​± ​12.9
BMI (kg/m^2^)	24.3 ​± ​3.2	23.0 ​± ​3.0	23.3 ​± ​3.0
Physical activity (PASE:/793) n ​= ​40	156.9 ​± ​83.7	201.6 ​± ​91.2	189.3 ​± ​90.4
KOOS n ​= ​38			
Symptoms (100 ​%)	88.3 ​± ​8.6	86.8 ​± ​12.4	87.2 ​± ​11.4
Pain (100 ​%)	89.6 ​± ​7.3	91.2 ​± ​13.9	90.7 ​± ​12.2
Stiffness subtotal (/8)	0.8 ​± ​1.5	1.2 ​± ​1.3	1.1 ​± ​1.3
Knee flexion ROM (degrees) n ​= ​40			
Right	148.5 ​± ​7.3	140.5 ​± ​9.5	142.7 ​± ​9.5
Left	145.4 ​± ​6.9	143.5 ​± ​7.1	144.0 ​± ​7.0
Mean	147.0 ​± ​6.6	142.0 ​± ​7.8	143.4 ​± ​7.8

Note: BMI: body mass index, F: females, KOOS: Knee Osteoarthritis Outcome Score, M: males, PASE: Physical Activity Scale for the Elderly, SD: standard deviation.

The pilot study data observed that participant-reported knee stiffness had reached statistical and meaningful levels by 90 ​min of prolonged sitting which increased up to 180 ​min of sitting (Fig. 2) (Stiffness at Baseline: 0.5 ​± ​0.8, 90-min: 3.0 ​± ​1.9, 120-min: 3.9 ​± ​2.2, 150-min: 4.4 ​± ​2.7, 180-min: 4.3 ​± ​3.0, p ​< ​0.005). Knee joint pain scores were also significantly different by 90 ​min of prolonged sitting compared with baseline (p ​≤ ​0.05) and remained stable for up to 3 ​h (Pain at Baseline: 0.5 ​± ​1.0, 90-min: 1.7 ​± ​1.8, 120-min: 2.0 ​± ​2.0, 150-min: 1.9 ​± ​2.3, 180-min: 1.8 ​± ​1.6), however pain remained under the MCID during the prolonged sitting protocol.

**Fig. 4:**
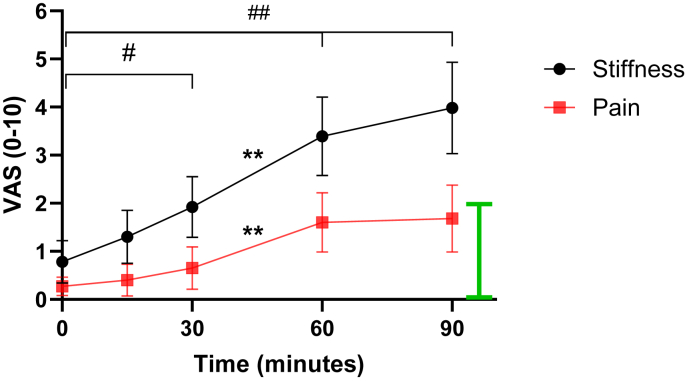
The time course of self-reported knee joint A) stiffness and B) pain via visual analogue scale (VAS) for up to 90 ​min of sitting with knees held at approx. 90°. Data are means and 95 ​% CI. Bars Denote difference from baseline at all timepoints for stiffness, #p ​< ​0.05, ##p ​< ​0.01 (Pain: baseline different at 60 & 90 ​min only, p ​< ​0.01, not shown on figure). Difference from previous time point for both stiffness and pain: ∗∗p ​< ​0.01. **Green bar** denotes Minimal Clinical Important Difference from VAS ​= ​0.

In the main study, higher levels of self-reported stiffness (n ​= ​31) when compared to baseline (0.8 ​± ​1.1) was observed after sitting for 30 (1.8 ​± ​1.6; p ​< ​0.04), 60 (3.4 ​± ​2.4; p ​< ​0.001) and 90 ​min (4.1 ​± ​2.6; p ​< ​0.001), as shown in Fig. 4. Additionally, a significant increase of self-reported pain (n ​= ​31) only occurred by 60 (1.4 ​± ​1.6; p ​< ​0.001) and 90 ​min of sitting (2.1 ​± ​2.1; p ​< ​0.001). The MCID in stiffness occurred by 60 ​min of sitting (+2.6 ​cm), while the MCID for pain was not reached over the 90-min sitting period.

**Fig. 5:**
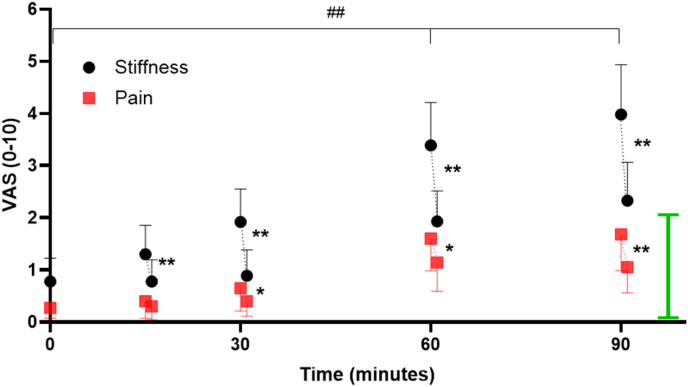
The 1-min post-sitting recovery of self-reported knee joint A) stiffness and B) pain via visual analogue scale (VAS) following up to 90 ​min of sitting with knees held at approx. 90°. Data are means and 95 ​% CI (only one direction shown for clarity). ## Denotes difference at recovery compared with baseline at 1-min post 60 & 90 ​min sitting only for stiffness (p ​< ​0.001) and pain (p ​< ​0.01). Difference from immediately post sitting time point to 1-min post-sitting recovery time point (indicated by dotted lines) for both stiffness and pain: ∗p ​< ​0.01, ∗∗p ​< ​0.01. **Green bar** denotes Minimal Clinical Important Difference from VAS ​= ​0.

The self-reported stiffness levels recorded 1-min after moving the knee joint following each prolonged sitting time point decreased significantly (Fig. 5, p ​< ​0.001), though stiffness recovered to baseline levels only after 15 and 30 ​min of sitting. After 60 and 90 ​min of sitting the 1-min post stiffness values were still significantly above baseline VAS levels (0.8 ​± ​1.2), at 1.9 ​± ​1.6 and 2.3 ​± ​2.0, respectively (p ​< ​0.001), though within MCID from baseline stiffness at all timepoints. The recovery of pain 1-min after moving the knee joint was significantly decreased after 30, 60 and 90-min of sitting (p ​< ​0.05; pain had not increased after 15-min sitting), with pain scores at baseline levels after 1-min post 15 and 30-mins of sitting. After 60 and 90-mins of sitting, the 1-min post stiffness values were still significantly above baseline levels (0.3 ​± ​0.5), 1.1 ​± ​1.5 (p ​< ​0.001) and 1.1 ​± ​1.3 (p ​= ​0.003), respectively, though all values were within the MCID.

**Fig. 6:**
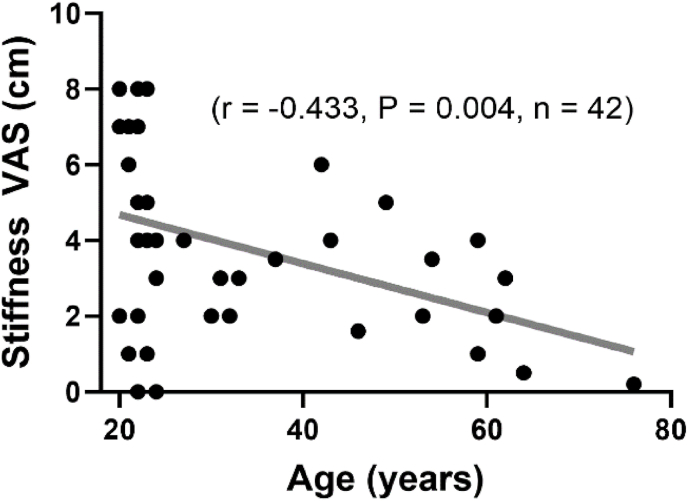
Correlation between self-reported knee joint stiffness after sitting for 90 ​min (VAS) with participant age.

A negative correlation between self-reported stiffness at 90-min sitting and participant age was observed (r ​= ​−0.433, p ​= ​0.004, n ​= ​42), see Fig. 6. Stiffness was also negatively correlated with KOOS-Symptoms (r ​= ​−0.472, p ​= ​0.003, n ​= ​38) and positively associated with pain at 90-min (r ​= ​0.645, p ​< ​0.001, n ​= ​42) and KOOS-Stiffness (r ​= ​0.433, p ​= ​0.007, n ​= ​38). However, no significant relationships were detected between stiffness at 90-min sitting with BMI, ROM, KOOS-pain, or PA (p ​> ​0.05). Pain at 90-min sitting was also negatively associated with age (r ​= ​−0.355, p ​= ​0.021, n ​= ​42) and KOOS-Symptoms (r ​= ​−0.361, p ​= ​0.026, n ​= ​38). Pain was not correlated with BMI, ROM, KOOS-pain, KOOS-Stiffness, or PA (p ​> ​0.05).

## 4 Discussion

The current study observed that healthy knee joint stiffness was perceived to rise by 30 ​min of sedentary seated time with participant's keeping their knees still, although the perceived joint stiffness did not reach a clinically meaningful level until 60 ​min of sitting. Joint pain had increased by 60 ​min of sitting, however never reached clinically meaningful levels. Both perceived knee stiffness and pain were able to be reduced within a minute by moving the knee joint even while still seated. Interestingly, lower stiffness levels after 90 ​min of sitting were associated with increased age in our healthy population.

The results of this investigation provide a time frame for stiffness onset in healthy populations, our cohort indicated an increase in perceived stiffness occurring after only 30 ​min of sitting. This finding is in line with a previous study which indicated no significant increase in knee stiffness in healthy adults after 20 ​min of sitting, but that had increased in people with PFP (+0.53/10 vs. +2.25/10) [29]. In comparison to the current study, their healthy participants were generally younger (22 ​± ​5 years). Future research could compare potential differences between joint conditions and explore pathological processes relating to the onset of knee stiffness and pain.

Encouragingly, in the current study, healthy individuals sitting still for prolonged periods, reported low perceived knee pain. Additionally, both joint stiffness and pain reduced with passive knee movement whilst remaining sitting. The importance of breaking up bouts of prolonged sitting has been well established [2] and includes decreased muscle stiffness [9] and improved cardiometabolic outcomes [7], fatigue and maintaining work productivity [11]. The current study highlights the clinical importance of establishing the timeline for stiffness to occur and reduce. This knowledge will assist health professionals, including Exercise Physiologists in exercise program scheduling and lifestyle recommendations. For example, in creating a schedule to move away from a sedentary work, lightly stretching or moving position when required to be sedentary, thus reducing joint stiffness during work or long-haul travel.

The current study observed an association with lower reported knee stiffness after 90 ​min of sitting with increasing age. Although this may appear counterintuitive, previous research has also reported that younger people with PFP were more likely to report problems with prolonged sitting [40]. As participants’ age so do previous life experiences, e.g. older individuals with healthy joints engaging in sedentary office work or long-haul travel and thus may find 15–90 ​min of sitting less symptomatic in proportion to an 8-h workday or 10-h flight. It would be interesting to observe correlation of stiffness after 90 ​min of sitting with age between healthy and early-stage OA symptomatic knees, with prolonged sitting being a risk factor for knee OA [41]. The current study did observe associations between stiffness and pain after sitting and with baseline KOOS scores. Previously it has been suggested that correlations between pain and stiffness in people with PFP could indicate that participants may misinterpret the two sensations [29]. The current study aimed to minimise this possibility by asking participants to report pain prior to moving the knee, followed by reporting stiffness during passive knee ROM afterwards. Additionally, there was no notable difference between knee stiffness and other secondary outcomes in the current study, likely due to including only healthy participants as changes in many of these secondary outcomes are commonly only perceived by pathological knees [27,42,43].

The sensations of stiffness and pain after sitting are likely driven by several factors. Limb immobilisation may cause synovial fluid to move less effectively [44,45], and surrounding tissues may become temporarily less flexible [46]. Circulatory pooling in the legs during sitting may add to the feeling of heaviness or stiffness [47]. Neurophysiological influences, including altered proprioception and reflexive co-contraction [48,49], could also heighten awareness of stiffness, particularly in younger adults who may have higher baseline neuromuscular tone. Pain, in contrast, may be linked to pressure on the kneecap joint or short-term reductions in blood flow around the knee [47,50]. Together, these factors help explain why stiffness develops quickly with sitting, improves with movement, and may be experienced differently across age groups.

The main strengths of the current research lie with our range of ages included and methodological rigor focusing on participant-reported and clinically relevant outcome measures. The main limitations of this pilot study include the issues regarding the self-reporting of symptoms, the use of both limbs to collect post-sitting measures and some missing data. Although joint stiffness is a key symptom of prolonged sitting and musculoskeletal pathologies, it is predominantly measured through patient-reported symptoms, which may limit its applicability. There are a range of mechanical and patient-reported outcome measures in use, highlighting the need for further research in this area [27]. The 10-cm Visual Analogue Scale (VAS) was used in the current study as a validated, reliable tool for capturing acute, single time point responses of stiffness and pain, consistent with OMERACT–OARSI recommendations for patient-reported outcomes [[51], [52], [53]]. And although not co-designed, the outcome measures used represents standard musculoskeletal research practice. For participant comfort and time required sitting, we utilised both limbs. Analysis revealed that there were no differences in outcomes at baseline between limbs, though note that this may be perceived as a limitation. We attempted to correct these limitations through protocol design (randomisation of visit and limbs; Fig. 1), recruitment greater than the required sample size, including healthy people across a range of ages, as well as clear instructions to participants prior to baseline measurements.

The current research findings have important research and clinical implications for healthy and musculoskeletal conditions. Once a timeframe in clinical populations (e.g. OA) is established, further research can use the time for onset of stiffness to assess changes across a range of clinical markers, e.g. cartilage metabolism specific biomarkers. Additionally, clinicians and symptomatic individuals may benefit from the timeline observed in healthy adults in the current study as a recommendation to reduce their musculoskeletal symptoms. Exercise Physiologists may highlight this to their clients in exercise program scheduling and lifestyle suggestions. The findings are also useful for healthy adults. In a corporate setting, a to move away from prolonged sedentary work without taking PA breaks, and for all adults to undertake light stretching during and after long sedentary bouts, breaking up sedentary time with exercise and for providing movement recommendations for reducing sedentary related stiffness during long-haul travel.

In conclusion, knee joint stiffness begins by 30 ​min of sitting in healthy adults. However, minimal clinically important increase of stiffness only occurs by 60 ​min of sitting, while pain was significant but not meaningfully increased at 60 ​min of sitting. Furthermore, only brief passive movement whilst remaining seated is all that is required to decrease these stiffness and pain sensations. Interestingly, older adults were less likely to report higher stiffness and pain following prolonged sitting. These findings can be applied to practice through recommendations for light intensity range of motion movements whilst sitting every 15–60 ​min. Future directions with this research should use the time course from these healthy adult participants to guide research into the time course for knee stiffness to occur in other healthy populations and musculoskeletal conditions.

## Declaration of Generative AI and AI-assisted technologies in the writing process

The authors did not use AI in any aspect of the research or manuscript writing.

## Funding

No funding was received for conducting this study.

## Declaration of competing interest

The authors have no conflicts of interest to declare that are relevant to the content of this article.
